# Polyphenols in Plants: Structure, Biosynthesis, Abiotic Stress Regulation, and Practical Applications (Review)

**DOI:** 10.3390/ijms241813874

**Published:** 2023-09-09

**Authors:** Natalia V. Zagoskina, Maria Y. Zubova, Tatiana L. Nechaeva, Varvara V. Kazantseva, Evgenia A. Goncharuk, Vera M. Katanskaya, Ekaterina N. Baranova, Maria A. Aksenova

**Affiliations:** 1 K.A. Timiryazev Institute of Plant Physiology, Russian Academy of Sciences, 127276 Moscow, Russia; mariia.zubova@yandex.ru (M.Y.Z.); nechaevatatyana.07@yandex.ru (T.L.N.); k.v.-90@mail.ru (V.V.K.); goncharuk.ewgenia@yandex.ru (E.A.G.); vera@katanski.com (V.M.K.); akse.masha@yandex.ru (M.A.A.); 2 N.V. Tsitsin Main Botanical Garden of Russian Academy of Sciences, 127276 Moscow, Russia; greenpro2007@rambler.ru; 3 All Russia Research Institute of Agricultural Biotechnology, Russian Academy of Agricultural Sciences, 127550 Moscow, Russia

**Keywords:** phenolic compounds, phenylpropanoids, flavonoids, antioxidant activity, environmental factors, regulation, health care

## Abstract

Phenolic compounds or polyphenols are among the most common compounds of secondary metabolism in plants. Their biosynthesis is characteristic of all plant cells and is carried out with the participation of the shikimate and acetate-malonate pathways. In this case, polyphenols of various structures are formed, such as phenylpropanoids, flavonoids, and various oligomeric and polymeric compounds of phenolic nature. Their number already exceeds 10,000. The diversity of phenolics affects their biological activity and functional role. Most of their representatives are characterized by interaction with reactive oxygen species, which manifests itself not only in plants but also in the human body, where they enter through food chains. Having a high biological activity, phenolic compounds are successfully used as medicines and nutritional supplements for the health of the population. The accumulation and biosynthesis of polyphenols in plants depend on many factors, including physiological–biochemical, molecular–genetic, and environmental factors. In the review, we present the latest literature data on the structure of various classes of phenolic compounds, their antioxidant activity, and their biosynthesis, including their molecular genetic aspects (genes and transfactors). Since plants grow with significant environmental changes on the planet, their response to the action of abiotic factors (light, UV radiation, temperature, and heavy metals) at the level of accumulation and composition of these secondary metabolites, as well as their metabolic regulation, is considered. Information is given about plant polyphenols as important and necessary components of functional nutrition and pharmaceutically valuable substances for the health of the population. Proposals on promising areas of research and development in the field of plant polyphenols are presented.

## 1. Introduction

A unique characteristic of plants, along with photosynthesis, is their ability to form various low-molecular-weight specialized (secondary) compounds that do not participate in primary metabolism [1,2]. The primary metabolites are crucial for plant growth and development, whereas secondary metabolites are viewed as essential components for their interaction with the environment [3,4].

The spectrum of synthesized secondary metabolites in plants is diverse. The main representatives are terpenes, alkaloids, cyanogenic glucosides, and polyphenols [1,4].

Polyphenols or phenolic compounds (PCs) are considered to be among the most prevalent secondary metabolites, synthesized in all plant cells [5,6]. Significant progress has been made to date in establishing their structure and chemical properties [7,8]. The biosynthesis of PCs is extensively studied, and the key enzymes participating in this process are identified [7,9,10]. Due to transcriptome research, there are data on genes that determine cells’ ability to produce these compounds [11,12], along with numerous molecular markers that are valuable for identifying phenol-producing plants [13].

Formerly, in the 20th century, PCs were considered only as a mechanism to eliminate high-carbon components from metabolism. However, now, their significant contribution to plant cell functionality is beyond doubt [3]. The production of these metabolites is essential for plant growth and development and protection against various biotic and abiotic factors [4,6,7].

The accumulation of PCs in the early stages of plant growth is known well [14]. The correlation between its formation and the process of photosynthesis [15], auxin metabolism [16], and cell protection against various stressors [7,9,17] has been proved. The latter characteristic is associated with the antioxidant capacity of PCs, which is determined by their chemical structure [7,18]. This aspect of the functional role of these specialized metabolites commands significant attention from researchers due to the broad-ranging activity of these metabolites within the human body. The PCs’ capillary-strengthening, antibacterial, antiviral, antitoxic, and neurodegenerative effects are known [19,20].

Plant-derived PCs, frequently referred to as bioflavonoids, are increasingly used as therapeutic pharmaceutical agents for treating diseases of various etiologies [4,21,22]. Despite the considerable number of publications regarding plant-derived PCs, their structure, biosynthesis, and function require further research.

This concerns the study of the structural diversity of these metabolites and their properties. A greater “detailing” of PCs’ biosynthesis is needed, including genes and transcription factors that ensure its functioning. Ideas about the influence of various environmental factors on the accumulation of PCs in plants are still rather contradictory and ambivalent. However, their study and evaluation are very important for agricultural crops and medicinal plants, which are successfully used as producers of biologically active compounds and nutraceuticals in the food industry and pharmacology.

In our review, we briefly present the current state of research on the structure of various PCs, their properties (mainly antioxidant activity), and the main stages of biosynthesis, including genes and transcription factors. In addition, we present recent data on the regulatory effect of various abiotic environmental factors (light, UV radiation, temperature, and heavy metals) on the accumulation of PCs in plants. Since these are some of the plant metabolites actively used in pharmacology, there is a small section on their use in medicine. The important fundamental and practical significance of PCs allowed us to consider the prospects for further research on these unique plant metabolites.

## 2. Polyphenols Structure, Properties and Antioxidant Activity

**Figure 1:**
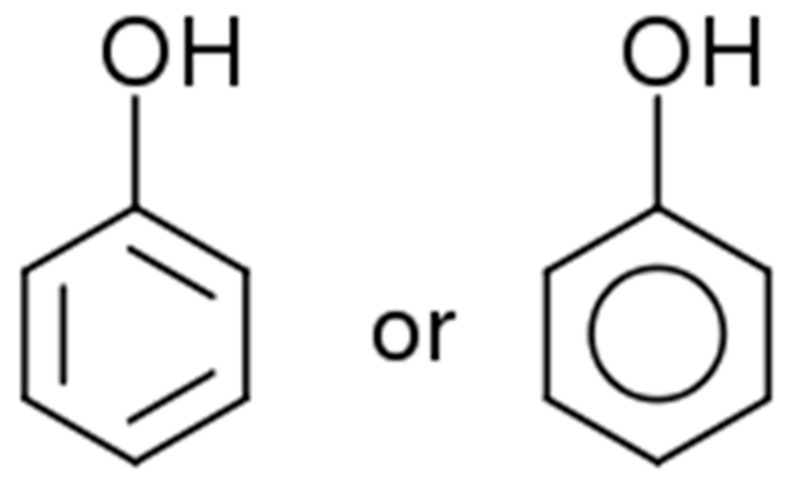
The structural formula of phenol.

Polyphenols are low-molecular-weight organic substances containing an aromatic (benzene or phenol) ring with one or more hydroxyl groups in their molecule [23]. It can be a simple compound or a complex polymer [24]. They are categorized into various classes and subclasses based on the chemical structure, the number of phenol rings, the position of functional groups, or the carbon skeleton [7,25,26]. Among them, phenol is the simplest and the least common form of PC in plants (Figure 1).

**Figure 2:**
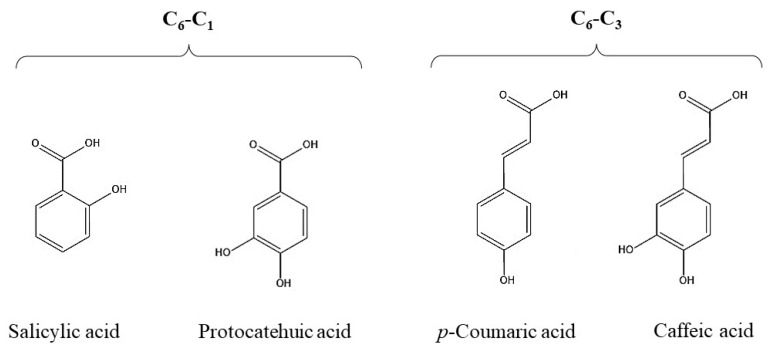
Structural formulas of simple polyphenols.

The presence of a single benzene ring along with a one-carbon or three-carbon side chain is a distinguishing feature of hydroxybenzoic and hydroxy-cinnamic acids, respectively (Figure 2). Their general formulas are usually denoted as C_6_-C_1_ and C_6_-C_3_, respectively [27]. It should be noted that they belong to the class of phenylpropanoids, which are widespread PCs in plants [9,10].

**Figure 3:**
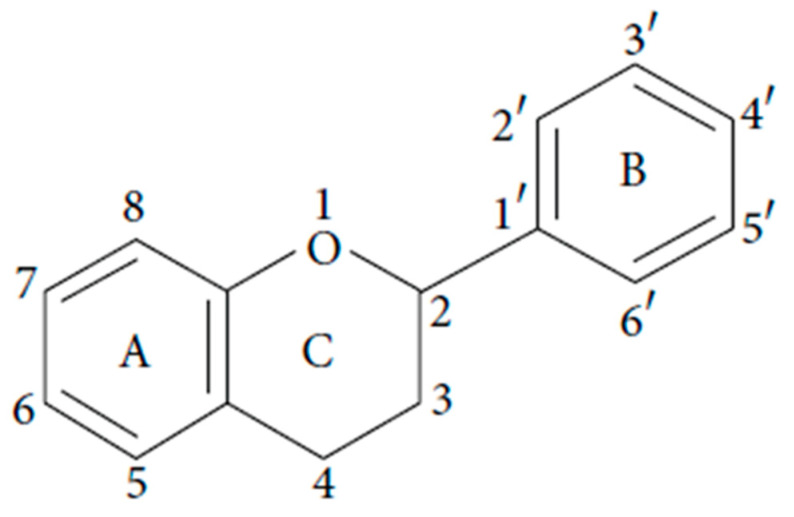
The structural formula of flavonoids.

Flavonoid-type PCs exhibit more complex structures, featuring two aromatic rings (labeled as A and B) interconnected by a three-carbon fragment (designated as C) (Figure 3). The general formula for flavonoids is denoted as C_6_-C_3_-C_6_.

**Figure 4:**
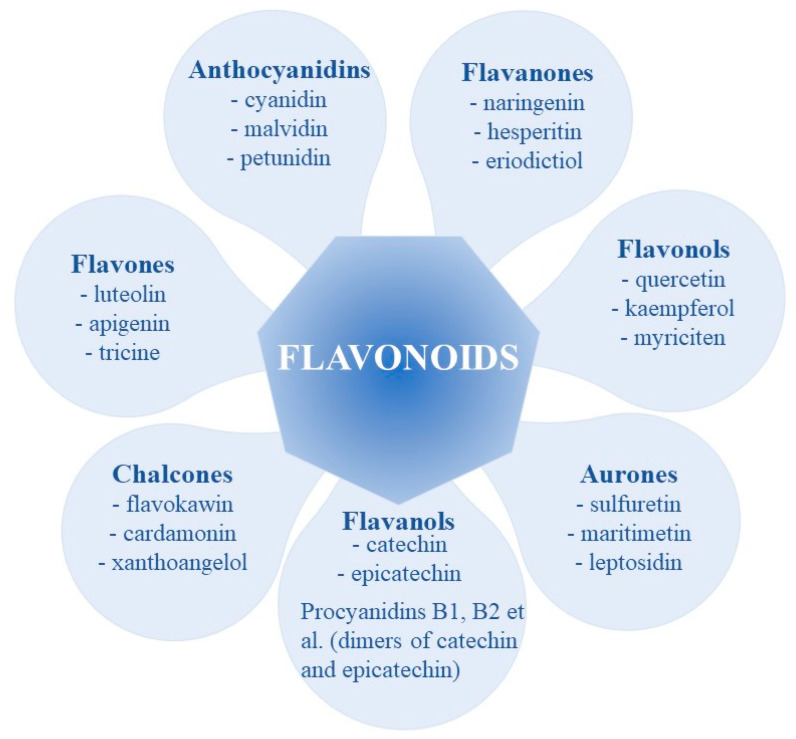
Main subclasses of flavonoids and some of their representatives.

Flavonoids are categorized into different subclasses depending on the degree of oxidation (or reduction) of the three-carbon fragment [8,26,27]. The main classes are shown in Figure 4.

**Figure 5:**
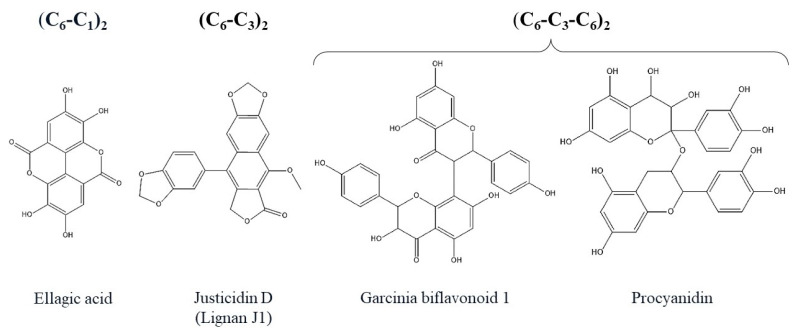
Structural formulas of dimers from different classes of polyphenols.

In addition to the monomeric PCs mentioned earlier, plants also produce oligomeric and polymeric forms. Oligomeric PCs include dimers of phenylpropanoids, flavones, and flavonols, as well as flavan-3-ols and (or) flavan-3,4-diols (depicted in Figure 5). The latter are known as proanthocyanidins [28].

**Figure 6:**
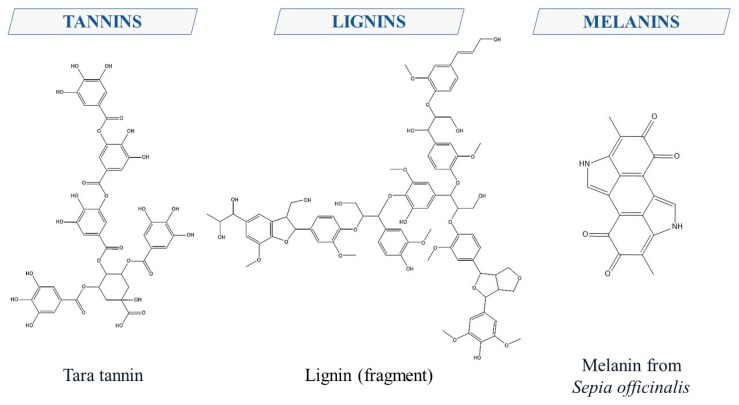
Structural formulas of polymeric compounds of phenolic nature.

Polymers of PCs are tannins, lignin, and melanins. Tannins are classified into hydrolyzable, condensed, thearubigins, and phlorotannins [27,29]. A unique phenolic metabolite formed from phenylpropanoid units is lignin, widely distributed in plant tissues [30,31]. Melanins, somewhat “conditionally” considered PCs since they are primarily formed through the acetate-malonate pathway, have also captured researchers’ interest due to their protective role not only in plants but also in humans [32,33,34]. Figure 6 showcases some of these substances.

At present, more than 10,000 PCs have been identified, including both water-soluble and organic-solvent-soluble or insoluble forms [23]. This number continues to grow due to the advancement and implementation of novel analytic research methodologies such as capillary electrophoresis, high-performance liquid chromatography, mass spectrometry, nuclear magnetic resonance, and others [25,26].

The properties of PCs are significantly influenced by their chemical structure [26]. Their ability to form hydrogen bonds (both intermolecular and intramolecular) relies on the degree of hydroxylation of the benzene ring and the position of the hydroxyl group (OH). For instance, *meta*-substituted diphenols (dioxibenzenes) exhibit significantly greater resistance to oxidation than *para*- and especially *ortho*-diphenols [35].

Due to the presence of hydroxyl and carboxyl groups in their molecules, PCs possess the ability to form conjugates with compounds such as sugars, organic acids, plant amines, and others [36]. In this process, glycosidic, methylated, methoxylated, and acylated compounds of a phenolic nature are synthesized. As indicated in earlier studies, flavonoids undergo hydroxylation at positions 3, 5, 7, 2, 3′, 4′, and 5′ (Figure 3). Additionally, glycosidic bonds form at positions 3 or 7, involving glucose, rhamnose, galactose, or arabinose [37]. Some researchers report that during the formation of polyphenolic glycosides, β-glycosidic bonds link one or more sugar residues (monosaccharides, disaccharides, and oligosaccharides) to the hydroxyl group (O-glycosides) or the carbon atom of the aromatic ring (C-glycosides) [38].

According to the literature [25], PCs are characterized by two primary properties: *reducing activity*, which governs their antioxidant properties and their sensitivity to oxidation, and *the binding properties*, which are attributed to their metal-chelating activities and their affinity for proteins, including enzymes, transport proteins, and receptors. It is these properties that determine the biological activity of these secondary metabolites in both plant and animal cells.

**Figure 7:**
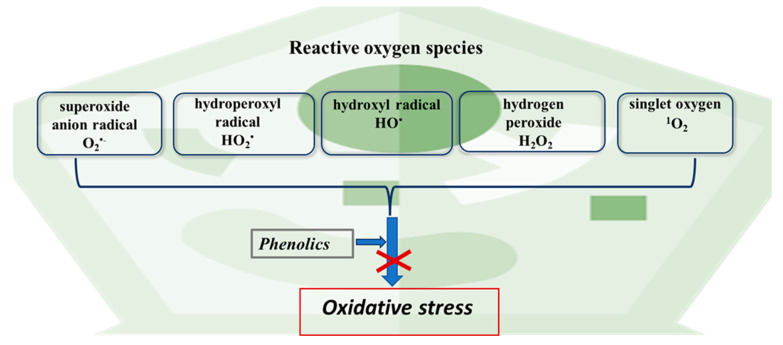
Polyphenols prevent the development of oxidative stress in plant cells induced by various reactive oxygen species.

The biological activity of PCs is frequently evaluated through their antioxidant properties [4,39]. These properties stem from their structural composition, comprising an aromatic ring, double bonds, and numerous functional groups [8,37]. PCs interact with reactive oxygen species (ROS) present in cells, which, at high concentrations, induce oxidative stress within them (Figure 7).

PCs scavenge hydroxyl radicals (OH) and superoxide anion radicals (O_2_) while also neutralizing active oxygen species such as hydrogen peroxide (H_2_O_2_) or singlet oxygen (^1^O_2_) [17]. As a result, they can prevent radical reactions instigated by these ROS, including lipid peroxidation, protein oxidation, oxidative damage to nucleic acids, alterations in the cytoskeleton, and other processes [18,40]. The antioxidant capacity of PCs is mainly determined by the number of hydroxyl groups in the molecule [7] as well as by the methylation and esterification of the compounds [35].

Some differences in the interactions between various plant PCs and ROS should be underlined. For instance, catechin gallates exhibit high activity against superoxide radicals exclusively (O_2_), while luteolin and kaempferol demonstrate activity against hydroxyl radicals (OH) [41]. The antioxidant activity of catechins is attributed to the ability of the hydroxyl groups of the catechol moiety to forge hydrogen bonds with the two oxygen atoms of lipid peroxide radicals [42]. The antioxidant activity of flavonoids (derivatives of luteolin and apigenin) isolated from celery leaves hinges on the location and quantity of -OH groups on the B-ring in their structures, as elucidated in a study by Wen et al. [43] based on data concerning DPPH• scavenging capacity and ABTS+• scavenging capacity. For some flavonoids, in particular quercetin and its glycosides, a higher activity of aglycones was noted [44,45].

The antioxidant activity of PCs in plants can also be attributed to their ability to chelate microelements; to inhibit enzymes involved in ROS formation, such as glutathione-S-transferase, microsomal monooxygenase, mitochondrial succinoxidase, NADPH oxidase, and xanthine oxidase; and to enhance the activity of high-molecular-weight antioxidants (enzymes) capable of scavenging radicals [26]. These mechanisms can operate independently or in specific combinations, which hinders their study [8,18].

While the formation of PCs is a characteristic feature across all members of the plant kingdom, their content and composition can vary significantly among different plant species and even within their respective organs [8,15,46]. In most instances, their accumulation remains below 1% of the dry weight [47,48,49]. However, there are exceptions, such as *Camellia sinensis*, where the PC content can surpass 20% of the dry weight [50]. It is noteworthy that the content of PCs tends to be higher, and their composition more diverse, in the above-ground parts of plants compared to their underground counterparts [51,52].

As previously mentioned, plants produce a wide variety of PCs with extremely diverse structural characteristics [8]. While flavonols are almost always present in chlorophyll-containing plant cells, they are rare in the absence of these organelles. In contrast, phenylpropanoids tend to exhibit relatively higher levels in such scenarios [8,46]. Based on these findings, we assume that the biosynthesis of PCs in plants is significantly influenced by the level of their intracellular differentiation and the functionality of chloroplasts, which are among the primary sites of their synthesis [15,53,54].

## 3. Biosynthesis of Polyphenols

The biosynthesis of PCs is a crucial component of plant secondary metabolism. The synthesis of their diverse structural forms occurs through two main metabolic pathways: the shikimate and aceto-malonate pathways [8,9,10].

**Figure 8:**
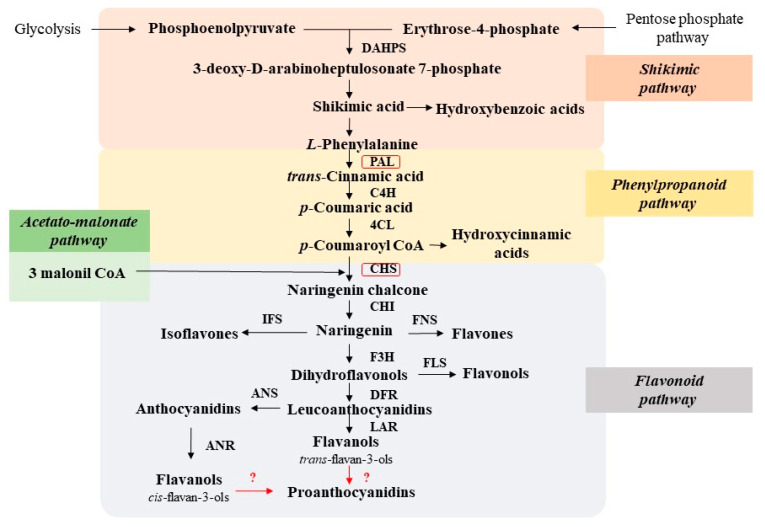
Biosynthesis pathway of phenolic compounds.

The name of the shikimate pathway originates from shikimic acid, which is the primary precursor in the biosynthesis of aromatic amino acids (*L*-phenylalanine, *L*-tyrosine, and *L*-tryptophan) as well as PCs [55,56]. Their substrates are products of primary metabolism: phosphoenolpyruvate from glycolysis and erythrose-4-phosphate from the pentose phosphate pathway (Figure 8). The 3-deoxy-D-arabinohexulose-7-phosphate, formed through their condensation, subsequently undergoes transformation into shikimic acid through a series of intermediate compounds catalyzed by enzyme-driven processes. As a result of additional transformations, shikimic acid can give rise to various hydroxybenzoic acids, such as *p*-hydroxybenzoic acid, protocatechuic acid, and gallic acid [10].

The predominant purpose of shikimic acid is to serve as a precursor for the synthesis of aromatic amino acids such as *L*-phenylalanine and *L*-tyrosine [9,10]. In this process, the deamination of *L*-phenylalanine by the key phenolic metabolism enzyme phenylalanine ammonia-lyase (PAL) results in the formation of *trans*-cinnamic acid [57]. This stage corresponds to the beginning of the phenylpropanoid pathway of PC biosynthesis. *Trans*-cinamic acid is regarded as one of the earliest phenolic metabolites produced in plant cells, acting as a bridge between the metabolism of aromatic amino acids and PCs [58,59]. The involvement of tyrosine ammonia-lyase in the transformation of *L*-tyrosine to *trans*-cinnamic acid has also been reported. However, its deamination is generally less pronounced compared to *L*-phenylalanine, which is the primary precursor for phenolic compounds [57]. It is suggested that in monocots, PAL can also function as *L*-tyrosine ammonia-lyase, acting as a bifunctional enzyme for both *L*-phenylalanine and *L*-tyrosine and converting *L*-tyrosine to *p*-coumaric acid (without the 4-hydroxylation step), albeit with reduced efficiency [58].

Enzymes: DAHPS, 3-deoxy-D-arabinoheptulosonate 7-phosphate synthase; PAL, phenylalanine ammonia-lyase; C4H, cinnamate 4-hydroxylase; 4CL,4-coumaroyl-CoA ligase; CHS, chalcone synthase; CHI, chalcone isomerase; F3H, flavanone 3-hydroxylase; FLS, flavonol synthase; FNS, flavone synthase; IFS, isoflavon synthase; DFR, dihydroflavonol 4-reductase; LAR, leucoanthocyanidin reductase; ANS, anthocyanidin synthase; ANR, anthocyanidin reductase.

A series of subsequent hydroxylations of *trans*-cinnamic acid leads to the formation of other hydroxycinnamic acids. Typically, these compounds do not accumulate in their free form within plant tissues, as they undergo further transformations. Among the most significant of them are *β*-oxidation resulting in hydroxybenzoic acids, reduction leading to cinnamic alcohols involved in lignin biosynthesis, the creation of various acyl derivatives and complex esters (such as chlorogenic acid), and the synthesis of coumarins [30,31,60]. This completes the formation of the major components in the phenylpropanoid pathway, which is a central component in the biosynthesis of natural PCs [61,62].

The aceto-malonate pathway plays a significant role in the biosynthesis of phenolic compounds [9,63]. In higher plants, this pathway is usually coupled with the phenylpropanoid pathway and leads to the formation of flavonoids [64]. Ring B in their structures is formed through the shikimate pathway (from hydroxycinnamic acid), while ring A is formed through the aceto-malonate pathway [63]. *p*-Coumaroyl-CoA and three molecules of malonyl-CoA are used as starting compounds (substrates), and their condensation leads to the formation of chalcone [65]. This reaction is catalyzed by chalcone synthase (CHS), the key enzyme in the biosynthesis of flavonoids [63,66]. The formed chalcone naringenin can easily be transformed into flavanone naringenin through the action of chalcone-flavanone isomerase (CHI). After undergoing changes in the oxidation state of the central heterocyclic ring of the molecule (due to redox reactions), naringenin can serve as a precursor for all other classes of flavonoids (flavan-3-ols, flavanones, flavones, anthocyanins, etc.) except for chalcones and dihydrochalcones. Enzymes involved in the formation of various classes of flavonoids have been extensively described in the literature [8,9,64,67].

Typically, most monomeric forms of phenolic compounds are not end products of phenolic metabolism; instead, they can participate in the formation of more complex oligomeric and polymeric structures. Among these are proanthocyanidins, which are derivatives of flavan-3-ols and are distributed in higher plant tissues [68]. Their biosynthesis occurs in the final stages of the flavonoid pathway, and the process of condensing flavan-3-ols into proanthocyanidins still remains unclear [69]. It is postulated that condensation can occur either through enzymatic pathways involving peroxidase, polyphenol oxidase, and laccase or through non-enzymatic means, specifically sequential autocondensation of flavan-3-ols [64,65,68].

In addition to biochemical studies on the biosynthesis of PCs, which remain relevant, the current emphasis is focused on studying the genes responsible for phenolic metabolism and their regulatory mechanisms. This shift is facilitated by advancements in molecular biology and functional genomics [13,59,70]. The field of genetic engineering is actively progressing to optimize plant phenolic profiles, with the primary aim of expediting the production of pharmacologically valuable compounds among other objectives [63,70,71].

More emphasis is currently being placed on studying gene expression related to the biosynthesis of PCs. For instance, gene networks responsible for regulating biochemical processes in *Camellia sinensis* have been documented [72,73]. Transcriptomic research has revealed metabolic pathways and crucial genes implicated in the biosynthesis, transport, and metabolism of catechins, caffeine, and L-theanine [74,75].

**Figure 9:**
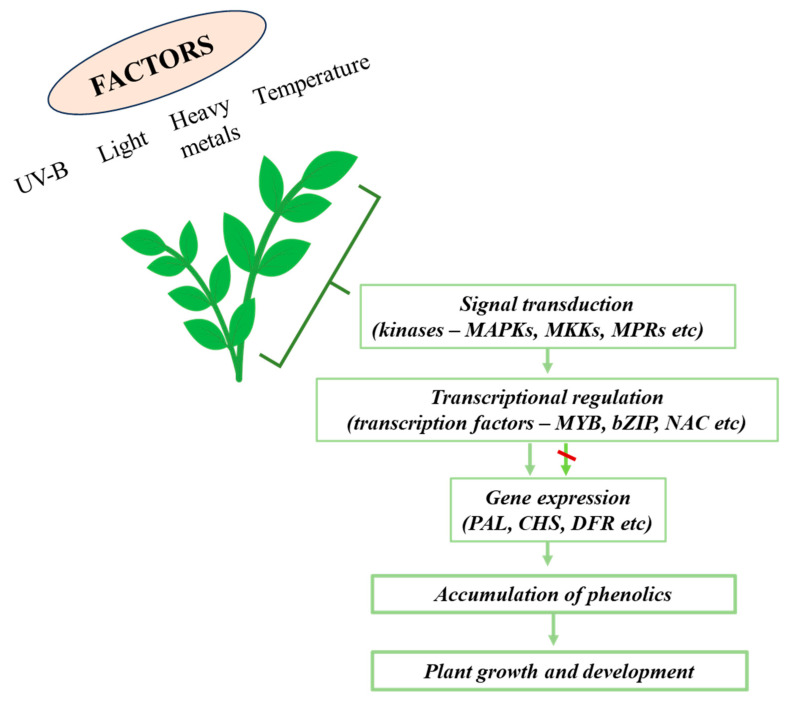
The effect of the abiotic factors on the activity of genes and transcriptional factors, regulating the polyphenol accumulation in plants.

Gene expression responsible for metabolite biosynthesis is under the control of various processes, including transcription, post-translational modifications, and micro-regulators like non-coding RNAs [58]. Among the key regulators of PC biosynthesis, transcription factors (TFs) stand out. TFs are DNA-binding proteins that bind to the promoter regions of target genes, modifying the rate of transcription initiation. In response to environmental signals (both internal and external, including phytohormones and abiotic factors), TFs can affect both structural and regulatory enzyme genes, thus influencing the accumulation of secondary metabolites (Figure 9).

Numerous families of TFs are known to be involved in phenolic metabolism, with one of the most prominent being the MYB TF family. They play a significant role in plant defense against various stresses [59,64]. It has been demonstrated that numerous MYBs play a regulatory role in the phenylpropanoid and flavonoid pathways within plants. Some of these TFs function as activators of enzyme genes, while others act as repressors [60]. For instance, the overexpression of the TF MusaMYB31 in bananas (*Musa* cultivar Rasthal) led to a reduction in the transcript levels of most genes within the phenylpropanoid and flavonoid pathways [76]. In contrast, studies conducted on poplar trees (*Populus* spp.) revealed that the TF PtMYB115 binds to the promoter regions of ANR1 and LAR3 genes, enhancing the expression of these genes and subsequently resulting in an increased accumulation of proanthocyanidins [77]. Furthermore, in grapevine (*Vitis vinifera*), the VvMYB5a transcription factor contributes to flavonoid synthesis by inhibiting lignin production. This unique regulatory mechanism helps maintain a balance in carbon flow between lignin and flavonoids in the plant [60]. Notably, a single MYB transcription factor can exert control over multiple genes within a pathway, and, conversely, a single gene can be subject to regulation by several MYB proteins [78].

Often, TFs are presented in the form of complexes. The MBW complex, composed of MYB, bHLH, and WD40 proteins, is a central transcriptional regulator in flavonoid biosynthesis [78]. This complex activates structural genes responsible for the flavonoid biosynthesis process, particularly anthocyanins. In several plants such as *Helianthus annuus* L., *Arabidopsis thaliana*, *Camellia sinensis*, *Narcissus tazetta*, *Medicago truncatula*, *Vitis vinifera*, and others, these transcription factors have been well characterized functionally [79].

Understanding the mechanisms of gene regulation in phenolic metabolism holds the potential to enhance the production of pharmacologically valuable secondary plant metabolites.

## 4. Abiotic Factors and Polyphenol Accumulation in Plants

**Figure 10:**
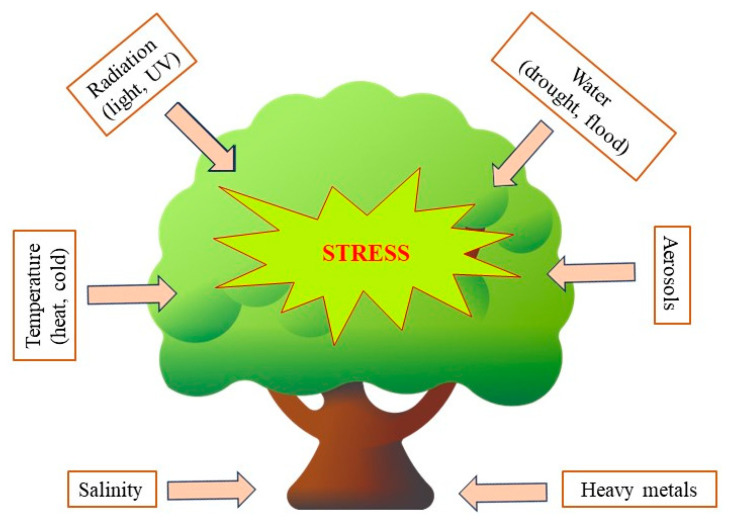
Environmental factors that can have a stressful effect on plants.

The interaction between plants leading a sessile lifestyle and their surrounding environment constitutes an indispensable prerequisite for their growth and development. However, fluctuating environmental conditions can exert stressful effects on them (Figure 10).

In these instances, the plant survival and preservation of their productivity and quantitative and qualitative characteristics, which are particularly important for agricultural and medicinal plants, depend on their adaptive potential [15,17,61]. The exploration of processes related to adaptation and resilience is one of the up-to-date domains within physiology, biochemistry, and molecular biology. These studies provide insights into the mechanisms of adaptation and facilitate the development of strategies to enhance plant resistance against exogenous influences. Notably, these strategies have proven effective in the selective breeding and development of transgenic stress-tolerant plants [71,79].

It is established that exposure to stressors leads to an excessive accumulation of reactive oxygen species (ROS) within plant cells. These ROS molecules interact with various biomolecules, including lipids, proteins, DNA, RNA, and other metabolites, exerting toxic effects that can ultimately result in cell death [80]. And in this case, an important role is assigned to antioxidants, including low-molecular-weight PCs. Their accumulation typically rises during exposure to stressful conditions, and this phenomenon is considered a benchmark for their resistance [2,40,81]. In the following sections, we will discuss these issues.

### 4.1. Light

**Figure 11:**
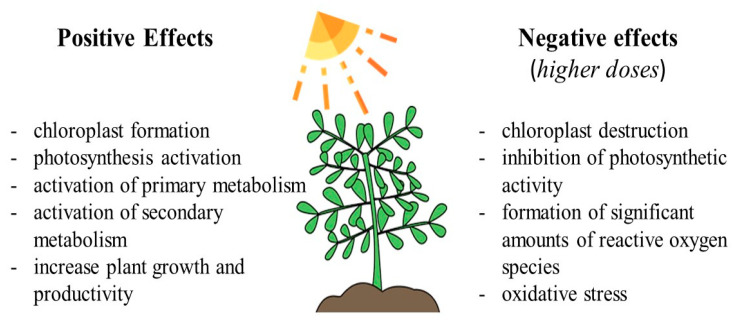
Light and its effect on plants.

Light is one of the “key” factors profoundly influencing plant life (Figure 11). This factor impacts many metabolic processes, including the biosynthesis of PCs [59]. Its regulatory role extends to numerous enzymes involved in phenolic metabolism, with their activation accompanied by the accumulation of these metabolites, primarily in their monomeric forms [9,15]. Additionally, light exposure contributes to the formation of chloroplasts, which represent one of the main sites for the biosynthesis of these secondary metabolites [53,54]. At the same time, light can also act as a stress-inducing factor, leading to an increase in the ROS levels in cells and even triggering an event termed an ‘oxidative burst’ [5].

The enhancement of the accumulation of phenolic antioxidants in plant tissues under light exposure has been discussed in several review articles [6,15]. The stimulatory effect of light on the levels of flavonoids, proanthocyanidins, and some other metabolites has been documented not only in leaves but also in in vitro cultures of *Camellia sinensis* [82,83]. Moreover, a discernible correlation has been established between the accumulation of proanthocyanidins and the expression of phenolic metabolism genes, encompassing PAL, flavanone 3-hydroxylase (*F3H*), flavonoid 3′-hydroxylase (*F3′H*), dihydroflavonol reductase (*DFR*), and anthocyanidin reductase1 *(ANR1*), predominantly observed within leaf tissues. Furthermore, a correlation between proanthocyanidin accumulation and the expression of phenolic metabolism genes, such as PAL, flavanone 3-hydroxylase (*F3H*), flavonoid 3′-hydroxylase (*F3′H*), dihydroflavonol reductase (DFR), and anthocyanidin reductase1 (*ANR1*), was observed in leaves. On the other hand, the formation of O-glycosylated flavonols correlated with the expression of chalcone synthase (*CHS*) and flavonoid 3′,5′-hydroxylase (*F3′5′H*).

Considerable emphasis is put on the investigation of how the spectral composition of light (red, far-red, blue, and green) influences the biosynthesis of PCs and their accumulation within plant tissues [5,6,15,59]. The stimulating impact of blue light on the production of these metabolites has been documented across a diverse array of plant species, including *Brassica napus, B. campestris* L. ssp. *chinensis* var. *communis*, and *B. oleracea* var. *alboglabra* Bailey [84,85]. Within callus cultures of *Camellia japonica*, the highest accumulation of PCs, encompassing flavonoids, was observed under the combined influence of red and blue light or, alternatively, blue and green light [86]. The involvement of MYB transcription factors in the light regulation of PC biosynthesis has also been reported [11]. In tartary buckwheat plants (*Fagopyrum tataricum*), a novel transcription factor has been identified and characterized, SG7 R2R3-MYB—FtMYB6, the promoter of which becomes induced by light [87]. Based on the acquired data, FtMYB6 stimulated the activity of FtF3H and FtFLS1 promoters while suppressing that of the Ft4CL promoter, thereby fostering the biosynthesis of flavonols within plant cells.

Overall, these divergences in the plant’s responsive reactions to light and/or its spectral composition are rooted in the functionalities of specific photoreceptors, attuned to distinct regions of the light spectrum [15,59]. Different classes of photoreceptors perceive wavelengths corresponding to blue (B, 445–500 nm), green (G, 500–580 nm), red (R, 620–700 nm), and far-red (FR, 700–775 nm) light. Their functional activity is regulated by the intensity and duration of the light exposure [88]. It implies that there is potential for regulating photomorphogenetic and biochemical processes, encompassing the accumulation of phenolic bioantioxidants, through artificial lighting as an economically valuable approach in industrial plant cultivation.

The presence of a significant number of studies on the effect of light on the accumulation of PCs in plants, their biosynthesis, and their gene regulation (some of them are presented in this review) does not yet allow us to obtain an accurate answer about the mechanism of its action. So far, we only have information about the response of different plants (species and varieties), which, in some cases, differs significantly. These differences are due to their physiological state, growing conditions, intensity and duration of light exposure, and other environmental factors. Consequently, biochemical and molecular genetic aspects of PC biosynthesis in plants exposed to light can be considered one of the promising and important areas of plant biology.

### 4.2. UV Radiation

One of the prominent challenges of our time is the shifting climate conditions across the planet. Within this context, particular attention is drawn to the effects of increased doses of solar radiation, including its ultraviolet (UV) range [89]. The intensity of this radiation is contingent upon the quantity and composition of anthropogenic emissions of greenhouse gases (carbon dioxide, methane, and nitrous oxide), cloud formation dynamics, and the extent of sea ice and snow cover on the Earth’s surface.

**Figure 12:**
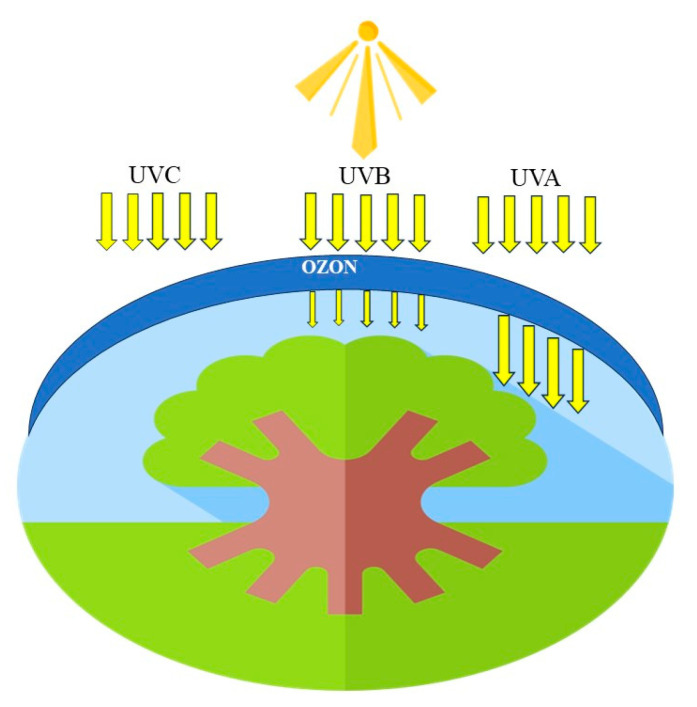
Ranges of UV light and its penetration through the ozone layer of the atmosphere.

UV light is categorized into distinct ranges: UV-A (315–400 nm), UV-B (280–315 nm), and UV-C (200–280 nm). Notably, UV-C does not reach the Earth’s surface, whereas UV-A fully penetrates the ozone layer (Figure 12). UV-B, accounting for 5% of the total UV radiation, is considered one of the most potent stressors for the vitality of numerous organisms [61,90].

Elevated levels of UV-B radiation are commonly observed in high-altitude regions and in areas with ‘ozone holes’ that form due to a reduction in stratospheric ozone concentration [91,92]. Exposure to this factor results in alterations in the morphophysiological, biochemical, and genetic characteristics of plants, the manifestation of which is contingent upon the ‘dosage’ of this stress factor [59,93]. Frequently, notable instances of their growth retardation, decreased productivity, reduced photosynthetic pigment content and intensity, the activation of mutagenic processes, and the disruption of DNA structure were observed [7,94]. In addition, the ‘ultimate’ biological effect of UV-B radiation exhibits not only a rapid response but also a temporally distant ‘outcome,’ attributed to the transformation of the de novo biosynthesis of various cellular components [95].

Despite all of these alterations, plants, as a whole, exhibit greater resilience to UV-B radiation compared to microorganisms and animals. This phenomenon could be attributed to the formation and functioning of adaptive mechanisms, including the accumulation of “protective” substances [61,94]. Among them are natural phenolic antioxidants, which, in this context, not only deactivate reactive oxygen species but also absorb the short-wavelength portion of solar radiation. As a result, they provide both physical and metabolic protection to cells against the damaging effects of UV-B radiation [89,90].

In most cases, exposure to UV-B rays increases the content of various phenolic metabolites within plant cells. One particularly striking illustration of this phenomenon was observed in a cell culture of rose (*Rosa damascena*) grown in in vitro conditions, where the accumulation of flavonoids increased by nearly 15 times [96]. Following exposure to UV-B radiation, the levels of flavonoids within olive leaves, such as 4′-methoxylutelin and 4′- or 3′-methoxylutelinglucoside, experienced an increase. Moreover, the phenylpropanoid *β*-hydroxy-verbascoside (a derivative of hydroxycinnamic acid) emerged [97]. Against the backdrop of changes in PC accumulation, the activation of phenolic metabolism genes occurred. For instance, in *Mangifera indica*, 3 out of the 21 chalcone synthase genes (MiCHS4, MiCHS1, and MiCHS17) were triggered in response to UV-B radiation exposure [98].

In conclusion, it is crucial to emphasize that the effects of increased UV-B radiation doses are contingent upon the endogenous content of PCs in plants, their composition, and their compartmentalization within plant tissues. This has been elucidated in a range of reviews [92,97,99].

### 4.3. Temperature

The growth and development of plants occur across a diverse temperature range [9,61]. However, significant fluctuations in temperature can impose stress, affecting physiological and biochemical processes, thereby leading to growth inhibition and developmental disturbances [100]. In this case, PCs play a crucial role in protecting plants from unfavorable temperature conditions.

It is well established that exposure to cold stress frequently triggers a heightened accumulation of anthocyanins in plants [101]. At the same time, an increase in the expression of genes responsible for flavonoid biosynthesis has also been noted. Particularly, in the case of *Brassica rapa*, a close correlation has been established between the plant resilience to cold stress and the expression of genes encoding dihydroflavonol-4-reductase (BrDFR) and anthocyanidin synthase (BrANS) [16,102]. Furthermore, it has been reported that exposure to lowered temperatures in *Malus sieversii* leads to an enhanced accumulation of anthocyanins, a phenomenon attributed to the involvement in this regulatory process of the transcription factor MdMYBPA1 [65]. The accumulation of anthocyanin in purple Chinese cabbages under low-temperature conditions was mediated through the induction of the regulatory genes BrTT8 and BrMYB2. These, in turn, triggered the activation of almost all of the late-stage biosynthesis genes responsible for these phenolic metabolites (BrDFR1, BrANS1, BrUGT79B1, DrUGT75C1, and Br5MAT) [103].

Lignin, a phenolic polymer, stands as one of the most prevalent forms of polyphenols in plant organisms [104]. The accumulation of lignin is a typical trait in most plant cells and, to a certain extent, contributes to their resilience to low temperatures. It has been reported that under cold stress conditions, its content within the epidermal cell layer increased [31]. This process facilitated the subsequent lignification of the cell wall, which “protected” intracellular contents from freezing and reduced cell damage during dehydration induced by freezing. Furthermore, there is evidence of the significant involvement of the transcription factors C2H2Zn and MYB in the biosynthesis of lignin during periods of abiotic stress exposure [105].

Changes in temperature during the vegetative phase of plants are frequently manifested by an increase, thereby affecting the plants’ antioxidant system and accumulation of PCs [17]. Through the example of *Solanum lycopersicon,* it was demonstrated that flavonols reduce the accumulation of reactive oxygen species induced by high-temperature stress [106]. Under elevated temperatures, an increase in the content of flavonoids and phenylpropanoids *in Glycine max* was observed [107]. It should also be noted that the activation of these processes may vary throughout the day and night. Through the example of grapevine (*Vitis vinifera*), it has been demonstrated that in darkness, with the temperature maintained at 35 °C, no changes in the quantity of flavonols were observed. However, at a more extreme temperature (45 °C), it decreased both during the night and daytime [108].

Molecular and genetic studies on chrysanthemum (*Chrysanthemum* cultivar ‘Fencui’) revealed a novel atypical transcription factor of subgroup 7 (SG7) R2R3-MYB (CmMYB012). This factor was induced in response to prolonged high-temperature exposure and inhibited flavonoid biosynthesis [109]. Moreover, it was demonstrated that it exerts an inhibitory effect on anthocyanin biosynthesis by suppressing the expression of CmCHS, CmDFR, CmANS, and CmUFGT.

An analysis of the literature reveals diversity in plant responses to temperature stimuli in terms of PCs: low temperatures generally lead to the activation of their biosynthesis, particularly anthocyanins, whereas high temperatures suppress this process [103,109].

All of this indicates that changes in the temperature of the surrounding environment influence the biosynthesis of various classes of PCs in plant tissues, and this process is dependent on the functional activity of genes responsible for their synthesis.

### 4.4. Heavy Metals

Among the abiotic stressors are heavy metals, a result of human technological activities, which mostly exert a negative impact on plant growth and metabolism [110]. The toxicity threshold of heavy metals hinges on the chemical characteristics of individual representatives, the form of their presence in the soil solution or other substrates, and the concentration and duration of exposure [111,112]. Certain heavy metals, including zinc, copper, and molybdenum, at elevated concentrations exhibit toxicity to plants, whereas at low levels, they play a vital role in supporting essential physiological processes. Heavy metals not involved in metabolic processes, like cadmium, lead, and mercury, can be toxic even at low levels [113].

Exposure to heavy metals is responsible for interrupting homeostasis in plants, which is mediated by increasing ROS levels in their cells [110]. But, in spite of that, plants keep surviving due to the functionality of antioxidant systems, where key positions belong to direct-acting antioxidants [13,114]. Among them are low-molecular-weight compounds, including PCs, which, in addition to neutralizing ROS, also act as agents chelating with heavy metals, thereby inhibiting metal-catalyzed free radical oxidation reactions [18,115]. Additionally, the ability of PCs to interact with the HM arises from the high nucleophilicity of their benzene rings and depends on the number and location of hydroxyl groups [7]. Flavonoids, the predominant class of PCs, play a crucial role in the chelation process with heavy metals. It has been revealed that in *Gynura pseudochina* plants, many of them are capable of chelating zink and cadmium, while catechins can chelate iron [116].

In most cases, plants’ exposure to heavy metals leads to an increased accumulation of various PCs within them. This is supported by data on the accumulation of catechins and quercetin in the roots of *Pínus* and *Zea mays* plants [7,117]. Callus cultures of *Amaranthus caudatus* and *Ginkgo biloba*, when exposed to copper, exhibited an increase in the content of flavonoids [19,118].

On the other hand, there are reports of a reduction in the content of PCs in plant tissues following exposure to heavy metals. The excess nickel in the surrounding environment led to a reduction in anthocyanin levels in the sprouts of *Lactuca sativa* [119]. A decrease in the levels of secondary metabolites in certain plants of the Asteraceae family has been reported under the conditions of metal-induced stress [120]. The absence of PC accumulation is attributed to the damaging effects of high levels of heavy metals, hindering the antioxidant system’s functionality and limiting the organisms’ ability to biosynthesize these metabolites.

The PC content in the plant cells is attributed to the functional activity of enzymes involved in their biosynthesis. Thus, in the sprouts of the red cabbage exposed to copper, along with an increased content of PCs, phenylalanine ammonia-lyase levels were augmented, which is responsible for the initial stages of phenolic metabolism [121]. In wheat, the activity not only of phenylalanine ammonia-lyase but also of tyrosine ammonia-lyase increased under elevated concentrations of lead and copper, and this effect was more pronounced under copper-induced stress [122].

The regulation of secondary metabolite formation in plant cells is strongly influenced by changes in the transcriptional levels of genes involved in their biosynthesis, a phenomenon directed by transcription factors (TFs), including the MYB family, the largest among them [11]. Within the latter, sub-family R2R3MYBs is the most interesting, which is involved not only in the ontogenetic development processes but also in the plants’ reactions to the stress conditions. This activity is mediated by the regulation of the biosynthesis of secondary metabolites, including flavonoids and monolignols [123]. It is established that this process occurs through the MBW (MYB-bHLH-WDR) complex, both for flavonoids and anthocyanins [61]. In plants of sweet wormwood (*Artemisia annua*), the overexpression of genes HMGR, ADS, CPYA171, and FDS enhances the formation of artemisinin against the backdrop of the toxic influence of copper and silver. Additionally, the elevated activity of genes PAL and CHS leads to an increase in concentrations of anthocyanins and flavonoids [116]. Under metal-induced stress, the transcription levels of genes encoding enzymes of the phenylpropanoid pathway (phenylalanine ammonia-lyase, chalcone synthase, shikimate dehydrogenase, and cinnamyl alcohol dehydrogenase) are linked to an increase in the content of PCs in plant tissues [61].

All of the aforementioned evidence suggests that the enhanced plants’ capability of biosynthesis, i.e., their capacity to accumulate PCs under the influence of heavy metals, is ensured by the functioning of various mechanisms critical for the survival and competitiveness of these organisms under abiotic stress conditions. Herewith, the activation of the phenylpropanoid pathway of PC biosynthesis often correlates with an increase in plant resistance and the manifestation of PCs’ functions such as antioxidant and protective functions. All of that is crucial for enhancing the quality of plant products used in the medical, pharmacological, and nutriceutical industries while developing a strategy for public health protection.

## 5. Phenolic Bioantioxidants in Public Health Protection

**Figure 13:**
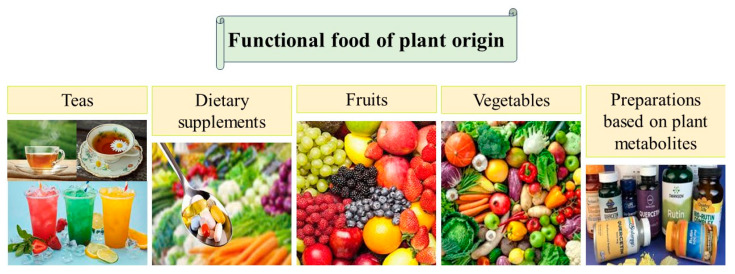
Basic plant foods for functional nutrition and maintaining human health.

One of the actively developing approaches for preserving and maintaining human health is the development of functional nutrition, along with the increasingly broader utilization of natural remedies [51]. This can reduce the usage of synthetic pharmacological products; enhance human vitality, including resistance to stress factors; and augment longevity (Figure 13).

Over the past few decades, significant attention worldwide has been devoted to the search for effective natural antioxidants that protect living organisms from the damaging effects of ROS [4]. Among them, PCs hold a distinctive position, demonstrating robust antioxidant capacity and potent antimicrobial, antiviral, antiatherosclerotic, and antihypertensive effects [124,125].

While the presence of ROS is an integral part of the normal functional activities of various organisms, the “uncontrolled” production of these molecules triggers oxidative stress, ultimately leading to the development of many diseases in humans [126]. In this scenario, PCs assume a vital and imperative role in protecting and preserving public health. Their aromatic nature and highly conjugated bond system with hydroxyl groups render these metabolites excellent donors of electrons or hydrogen atoms [18]. The mechanism of antioxidant action of PCs includes their reductive capacity in neutralizing ROS, as well as their ability to chelate metal ions that trigger oxidative stress. Furthermore, they can inhibit enzymes participating in ROS formation and activate antioxidant enzymes [26].

It is important to emphasize that PCs are not synthesized within the human body but rather enter it through food chains while retaining their inherent antioxidant activity [127,128]. This is precisely why pharmacologists and other experts show significant interest in studying plants as potential “sources” of phenolic phytonutrients with diverse biological activities (Table 1).

Antimicrobial, antiviral, antiatherosclerotic, capillary-strengthening, and anticancer effects are characteristic both of plant extracts and specific natural PC representatives [124,125,136]. According to preclinical and clinical research, the prolonged consumption of a diet rich in PCs (such as quercetin, resveratrol, gallic acid, and caffeic acid) reduces the incidence of cardiovascular diseases, diabetes, cancer, and atherosclerosis and also provides protection against certain types of allergies while slowing down the progression of Alzheimer’s disease [4,127]. Reports have indicated the involvement of these plant metabolites, including flavonoids, in activating the immune response of human cells to coronavirus infection (SARS-CoV-2) [137]. It is also worth mentioning that under specific conditions (alkaline pH and high metal content), polyphenolic compounds can act as pro-oxidants. This property contributes to their anticancer activity, as they can inhibit the proliferation of cancer cells [138].

Compounds of a phenolic nature are widely distributed in fruits, vegetables, grains, nuts, spices, tea, and other crops, with their quantity varying significantly. For instance, the total concentration of polyphenols in black-eyed peas can reach 1200 (mg-eq. gallic acid/g dry weight); in pomegranate leaves, 199.26 (mg-eq. gallic acid/g dry weight); and in peppermint, 70.06 (mg-eq. gallic acid/g dry weight) [48,134,135].

Including plant-based foods with high phenolic antioxidant content in the diet can help prevent oxidative stress in the body [48,139]. However, consuming PCs in the form of high-dose bioactive supplements may lead to adverse side effects in individuals [140]. This also applies to the use of dihydroquercetin, a commonly used capillary-strengthening agent, as excessive doses can result in various metabolic disruptions within the body [141].

It is worth highlighting that PCs in plants primarily accumulate in a conjugated form (as glycosides, acylglycosides, etc.) [142]. After their entry into the human body, they undergo various transformations in the digestive tract, including deglycosylation, methylation, sulfation, and glucuronidation [124,143]. An important role in the bioavailability of PCs belongs to the microbiome of the large intestine, which is involved in the cleavage of flavonoids to phenolic acids, which contributes to their reabsorption. It has been shown that the digestibility of PCs, including flavonoids, depends on individual characteristics of a person, such as gender, age, the presence of pathologies, and genetics [8,144]. The intake of plant-derived PCs into the human body, their transportation, and their pharmacological activity have been discussed in a series of reviews in recent years [31,145,146].

The effectiveness of the prevention and treatment of human diseases when eating plant foods enriched with PCs is beyond doubt. However, they are used not only fresh but also after various treatments (heating, cooling, preservation, drying, and fermentation). In this case, the composition, content, and biological activity of PCs depend on the type of process, the duration of exposure, the intensity of the regime, and the class of these secondary metabolites. All of this can increase the bioavailability of PCs for the human body due to their structural changes [147,148].

To overcome the problem of PC bioavailability, systems for their delivery using biocompatible materials such as nanoparticles, including liposomes, phytosomes, lipid and protein nanoparticles, micelles, and natural and synthetic polymers, have been developed [149]. They allow the protection of phenolic compounds from degradation; improve their solubility, cellular absorption, and stability; and maintain pharmacological activity in the human body. For example, nanocubasomas with anthocyanin-rich *Cornus mas* extract had a size of 22.75 nm. This contributed to their good entry into the cells. The stability of anthocyanins in this delivery system was 92%, while in the free extract, it was 75% [150].

Despite the pharmacological activity of many PCs and their effectiveness in the prevention and treatment of a number of diseases, the commercial production of these secondary metabolites is limited. This is due to the limited nature of the plant materials used to obtain them and the difficulty in isolating individual components [151]. An important limiting factor is the development of effective “delivery systems” of PCs into the human body that ensure their bioavailability [152]. We should not forget about the effect of high doses of PCs, which are not always optimal for maintaining human life. All of this requires further scientific and clinical studies to establish a safe dosage of pharmacological preparations containing these metabolites, improve their bioavailability, and develop the foundations for the effectiveness of both extracts of plant materials and individual compounds.

In conclusion, it should be noted that PCs are valuable phytonutrients, contributing to the prevention and/or treatment of a broad spectrum of human diseases as well as protecting the body from various stressors. Their “multifaceted” impact (with over 40 types of biological activity), non-toxicity, and widespread distribution in functional foods and medicinal plants allow us to view these secondary metabolite compounds as factors that modify the biological response of the organism. They hold significant importance in protecting public health all over the world.

## 6. Conclusions and Future Perspectives

PCs are unique secondary metabolites that play a role in numerous physiological and biochemical processes within plants. They also exhibit high antioxidant activity, which has led to their successful utilization in pharmacology for treating diseases of various etiologies. The application of high-performance liquid chromatography, mass spectrometry, and other analytical methods has enabled the acquisition of new insights into the structure, properties, and biological activity of diverse constituents of phenolic metabolism. And this field requires further research due to the variety of chemical structures of PCs and the ability to form complexes with various metabolites. Biochemical and molecular-genetic research has refined our understanding of the various stages of PC biosynthesis, along with the enzymes, genes, and transcription factors engaged in these pathways. However, there is still a lack of clarity regarding the formation of proanthocyanidins, one of the prevalent PCs in plants that vary in their degree of polymerization. Our knowledge about the gene networks of phenolic metabolism and their functioning within plant cells is still insufficient. The examination and analysis of antioxidant activity in both plant extracts and individual phenolic compounds can be regarded as one of the actively developing areas in plant biology, physiology, and pharmacological medicine. In this context, the ecological aspect of these studies is of great significance, considering the substantial shifts in temperature regimes, light, and UV exposure and the accumulation of heavy metals on the planet. These changes may be a consequence of technological atmospheric pollution. All of these factors result in considerable changes in the life processes and productivity of plants, including the accumulation of phenolic bioantioxidants—crucial components for plant functional nutrition and the preservation of human health. The necessity of investigating the impact of these stress factors is beyond doubt. This research will allow us to “evaluate” the nature of their response and develop strategies to regulate the resistance and adaptation of plants to changing environmental conditions, including at the level of vital bioactive metabolites like PCs. Among the promising directions for further research is the production of nanoparticles, with PCs as important regulators of the vital activity of various organisms, including plants and humans. Despite significant progress in the study of plant PCs and their functional activity, there are still many unresolved issues that are of great interest to scientists of various specialties—chemists, biologists, geneticists, biotechnologists, pharmacists, physicians, and others. To a large extent, this is due to the regulation of the accumulation and composition of these biologically active metabolites in plants used for food and medicinal purposes for the health and preservation of the population.

## Figures and Tables

**Table 1:** Plant Polyphenols and Their Pharmacological Activity.

Class of Polyphenols	Phenolic Compound	Pharmacological Activity	Plant Source	References
Flavonols	quercetin, kaempferol, myricetin, fisetin	anti-inflammatory, immunomodulatory, antiviral (against SARS-CoV-2, influenza, HIV)	grapefruit, capers, parsley, elderberry, sorrel	[129]
Flavanes	epicatechin, epigallocatechin gallate, epigallocatechin	neuroprotective, anti-inflammatory, capillary-strengthening	tea plant, cranberry, strawberry, blackberry, kiwi, cherry, pear, avocado	[130]
Flavones	apigenin, luteolin, chrysin	antimutagenic, anticarcinogenic, anti-inflammatory	green bell pepper, thyme, parsley, spinach, celery, chamomile, orange	[131]
Anthocyanins	delphinidin, cyanidin, malvidin	antidiabetic, antimicrobial, neuroprotective, cardioprotective, anticarcinogenic	blueberry, cranberry, lingonberry, grape, eggplant	[132]
Flavanols	proanthocyanidins	cardioprotective, neuroprotective, immunomodulatory, antidiabetic, anticancer	rosehip, lingonberry, cranberry, elderberry, black elder, currant, persimmon, quince	[69]
Flavones	naringenin	anticancer, antiviral, antibacterial, cardioprotective, antidiabetic	lemon, orange, grapefruit, tomato	[133]
Phenylpropanoids	rosmarinic acid, chlorogenic acid	neuroprotective, anti-inflammatory, antimicrobial hepatoprotective, immunomodulatory, antidiabetic, antitumor	rosemary, mint, sage, tea plant, apple, artichoke, carrot	[134,135]

## Data Availability

The data presented in this study are available on request from the corresponding author.
